# Intermediate hyperglycaemia and 10‐year mortality in resource‐constrained settings: the PERU MIGRANT Study

**DOI:** 10.1111/dme.14298

**Published:** 2020-04-03

**Authors:** M. Lazo‐Porras, A. Ruiz‐Alejos, J. J. Miranda, R. M. Carrillo‐Larco, R. H. Gilman, L. Smeeth, A. Bernabé‐Ortiz

**Affiliations:** ^1^ CRONICAS Centre of Excellence in Chronic Diseases Universidad Peruana Cayetano Heredia Lima Peru; ^2^ School of Medicine Universidad Peruana Cayetano Heredia Lima Peru; ^3^ Division of Tropical and Humanitarian Medicine Geneva University Hospitals and University of Geneva Geneva Switzerland; ^4^ Autonomic Dysfunction Centre Department of Medicine Vanderbilt University Medical Centre TN USA; ^5^ Department of Epidemiology and Biostatistics School of Public Health Imperial College London London UK; ^6^ Department of International Health Bloomberg School of Public Health Johns Hopkins University Baltimore MD USA; ^7^ Faculty of Epidemiology and Population Health London School of Hygienel and Tropical Medicine London UK

## Abstract

**Aim:**

To determine whether intermediate hyperglycaemia, defined by fasting plasma glucose and HbA_1c_ criteria, is associated with mortality in a 10‐year cohort of people in a Latin American country.

**Methods:**

Analysis of the PERU MIGRANT Study was conducted in three different population groups (rural, rural‐to‐urban migrant, and urban). The baseline assessment was conducted in 2007/2008, with follow‐up assessment in 2018. The outcome was all‐cause mortality, and the exposure was intermediate hyperglycaemia, using three definitions: (1) impaired fasting glucose, defined according to American Diabetes Association criteria [fasting plasma glucose 5.6–6.9 mmol/l (100–125 mg/dl)]; (2) intermediate hyperglycaemia defined according to American Diabetes Association criteria [HbA_1c_ levels 39–46 mmol/mol (5.7–6.4%)]; and (3) intermediate hyperglycaemia defined according to the International Expert Committee criteria [HbA_1c_ levels 42–46 mmol/mol (6.0–6.4%)]. Crude and adjusted hazard ratios and 95% CIs were estimated using Cox proportional hazard models.

**Results:**

At baseline, the mean (sd) age of the study population was 47.8 (11.9) years and 52.5% of the cohort were women. The study cohort was divided into population groups as follows: 207 people (20.0%) in the rural population group, 583 (59.7%) in the rural‐to‐urban migrant group and 198 (20.3%) in the urban population group. The prevalence of intermediate hyperglycaemia was: 6%, 12.9% and 38.5% according to the American Diabetes Association impaired fasting glucose definition, the International Expert Committee HbA_1c_‐based definition and the American Diabetes Association HbA_1c_‐based definition, respectively, and the mortality rate after 10 years was 63/976 (7%). Intermediate hyperglycaemia was associated with all‐cause mortality using the HbA_1c_‐based definitions in the crude models [hazard ratios 2.82 (95% CI 1.59–4.99) according to the American Diabetes Association and 2.92 (95% CI 1.62–5.28) according to the International Expert Committee], whereas American Diabetes Association‐defined impaired fasting glucose was not [hazard ratio 0.84 (95% CI 0.26–2.68)]. In the adjusted model, however, only the American Diabetes Association HbA_1c_‐based definition was associated with all‐cause mortality [hazard ratio 1.91 (95% CI 1.03–3.53)], whereas the International Expert Committee HbA_1c_‐based and American Diabetes Association impaired fasting glucose‐based definitions were not [hazard ratios 1.42 (95% CI 0.75–2.68) and 1.09 (95% CI 0.33–3.63), respectively].

**Conclusions:**

Intermediate hyperglycaemia defined using the American Diabetes Association HbA_1c_ criteria was associated with an elevated mortality rate after 10 years in a cohort from Peru. HbA_1c_ appears to be a factor associated with mortality in this Peruvian population.

## What's new?

- Intermediate hyperglycaemia is an important risk factor for diabetes, but the proportion of people with this condition varies according to the definition used.
- The extent to which impaired fasting glucose, impaired glucose tolerance and/or HbA_1c_ criteria are associated with mortality remains unclear.
- Intermediate hyperglycaemia assessed using the HbA_1c_‐based American Diabetes Association definition was associated with increased mortality rate in a 10‐year cohort from a Latin American country.
- HbA_1c_ appeared to be a measurement associated with mortality in this Peruvian population.

**What's new?**

- Intermediate hyperglycaemia is an important risk factor for diabetes, but the proportion of people with this condition varies according to the definition used.
- The extent to which impaired fasting glucose, impaired glucose tolerance and/or HbA_1c_ criteria are associated with mortality remains unclear.
- Intermediate hyperglycaemia assessed using the HbA_1c_‐based American Diabetes Association definition was associated with increased mortality rate in a 10‐year cohort from a Latin American country.
- HbA_1c_ appeared to be a measurement associated with mortality in this Peruvian population.

## Introduction

Type 2 diabetes mellitus is a major public health burden: 80% of cases are in low‐ and middle‐income countries and the economic burden and mortality rate are higher in these settings than in high‐income countries [1]. Intermediate hyperglycaemia is an important risk factor for diabetes, but the proportion of people with this condition varies according to the definition used [2]. The relevance of the term ‘intermediate hyperglycaemia’ is therefore debated because of the arbitrary cut‐off points used in its definition [3].

The association between diabetes and mortality is well known and may vary according to age, cardiovascular disease, renal complications and glycaemic control [4]; however, there is debate regarding the association of intermediate hyperglycaemia and mortality. A systematic review and meta‐analysis including 53 cohorts, totalling more than 1.5 million individuals, found that intermediate hyperglycaemia definitions that use impaired fasting glucose (IFG), defined according to the American Diabetes Association (ADA) criteria [fasting plasma glucose (FPG) 5.6–6.9 mmol/l (100–125 mg/dl)] and WHO criteria [FPG 6.1–6.9 mmol/l (110–125 mg/dl)], or use impaired glucose tolerance [IGT, defined as 2‐h plasma glucose 7.8–11.0 mmol/l (140–199 mg/dl)] were associated with all‐cause mortality. This was not the case when the ADA HbA_1c_‐based [HbA_1c_: 39–47 mmol/mol (5.7–6.4%)] or the International Expert Committee (IEC) HbA_1c_‐based definitions of intermediate hyperglycaemia [HbA_1c_ 42–47 mmol/mol (6.0–6.4%)] were used [5]. In addition, a study in Taiwan found that IFG defined according to the WHO was associated with an increase in cardiovascular disease and/or diabetes mortality; however, using the ADA’s definition of IFG, the number of people was four times greater, but there was a lower mortality risk [6].

As far as we are aware, no study has reported on the association of intermediate hyperglycaemia with mortality in a Latin American country. However, recent studies have highlighted the higher mortality risk attributable to type 2 diabetes in Latin American countries compared to high‐income countries, as shown in a recent systematic review [7]. It is therefore important to assess whether impaired fasting glucose, impaired glucose tolerance or intermediate hyperglycaemia, defined according to HbA_1c_ levels, are associated with mortality in these populations. The results of the present study should contribute to the worldwide evidence of intermediate hyperglycaemia and its relevance as a predictor of mortality. The aim of this study was to determine the association of intermediate hyperglycaemia status, assessed using three different definitions (IFG based on the ADA criteria, the ADA HbA_1c_‐based definition and the IEC HbA_1c_‐based definition), with 10‐year mortality in a Latin American country. Additionally, we explored the associations of different definitions of type 2 diabetes with mortality.

## Methods

### Study design, setting and participants

A new analysis of the PERU MIGRANT Study was conducted to evaluate mortality 10 years after baseline. The baseline assessment was conducted in 2007–2008 and the follow‐up in 2018.

The study population was categorized into three groups: urban, rural‐to‐urban migrants, and rural. A single‐stage random sampling method was used in the three population groups and the sample was stratified by sex and age (30–39, 40–49, 50–59 and ≥60 years). Urban participants were born and currently lived in Pampas de San Juan de Miraflores, a peri‐urban shantytown in Lima, the capital city, during the baseline assessment. Rural participants were selected from the adult population permanently living in San Jose de Secce in Ayacucho (3239 m above sea level). Rural‐to‐urban migrants lived in Pampas de San Juan de Miraflores and were born in Ayacucho, Peru. There was no overlap between the three population groups as the selection criteria were based on two factors: (1) place of birth and (2) place of residence.

The PERU MIGRANT Study settings and the characteristics of the enrolled participants have been described in detail elsewhere [8].

### Follow‐up assessment

At the 10‐year follow‐up the vital status of participants (dead or alive) was recorded using their National Identifier Number, which was verified in the national records using the National Registry of Identification and Civil Status (*RENIEC*) information. For this assessment, surviving participants were not directly contacted as only vital status and date of death or censoring was required. If a participant had died, the date of death was used, if the participant was alive, then the date on which the search was conducted was considered as the censoring date.

### Variables definition

All‐cause mortality was the outcome of interest and was defined by the occurrence of any fatal event at any point during the 10 years of follow‐up. Vital status and exact date of death were obtained by reviewing records provided by *RENIEC*. In this follow‐up, data obtained from participants with a valid national identity document observable in the *RENIEC* system were assessed. Vital status was then categorized as ‘alive’ or ‘dead’, and the time between baseline and the date of death or censorship was estimated in years.

The exposure of interest was intermediate hyperglycaemia, measured at baseline, and assessed using the three different definitions: (1) ADA‐defined IFG [FPG 5.6–6.9 mmol/l (100–125 mg/dl)] [9]; (2) the ADA HbA_1c_‐based definition [HbA_1c_ 39–46 mmol/mol (5.7–6.4%)] [9]; and (3) the IEC HbA_1c_‐based definition [ HbA_1c_ 42–46 mmol/mol (6.0–6.4%)] [10]. Each definition also included a threshold above which participants were classified as having type 2 diabetes, and this group was added to those participants who self‐reported physician‐diagnosed type 2 diabetes and those receiving glucose‐lowering drugs (Table 1) [10].

**Table 1:** Definitions of normal glycaemia, intermediate hyperglycaemia and type 2 diabetes

	Normal glycaemia	Intermediate hyperglycaemia	Type 2 diabetes
ADA IFG‐based definition	FPG <5.6 mmol/l (<100 mg/dl)	FPG 5.6–6.9 mmol/l (100–125 mg/dl)	FPG ≥ 7 mmol/l (126 mg/dl)
ADA HbA_1c_‐based definition	HbA_1c_ <39 mmol/mol (5.7%)	HbA_1c_ 39–46 mmol/mol (5.7–6.4%)	HbA_1c_ ≥ 53 mmol/mol (6.5%)
IEC HbA_1c_‐based definition	HbA_1c_ < 42.1 mmol/mol (6.0%)	HbA_1c_ 42–46 mmol/mol (6.0–6.4%)	HbA_1c_ ≥53 mmol/mol (6.5%)

ADA, American Diabetes Association; FPG, fasting plasma glucose; IEC, International Expert Committee; IFG, impaired fasting glucose.

Other participant characteristics, measured at baseline, that were included in the analysis as potential confounders were age (four categories: 30–39, 40–49, 50–59 and ≥ 60 years), gender (male and female), and education level (< 7 years and ≥7 years). Socio‐economic status was assessed through a wealth index based on the possession of household resources and household facilities (radio, types of television, refrigerator, computer, telephone, mobile phone, internet, cable television, motorcycle, car and gas cooker), and was divided into tertiles (low, middle and high socio‐economic status). Population group included rural, rural‐to‐urban migrant and urban categories. Current daily smoking (self‐reported smoking of at least one cigarette per day), alcohol intake (self‐reported low or high consumption based on the number of bottles of beer or equivalent), and physical activity levels, were also assessed. The latter variable was categorized according to the International Physical Activity Questionnaire as high/moderate if the participant performed ≥5 days of any combination of walking, moderate‐intensity, or vigorous‐intensity activities achieving at least 600 metabolic equivalent of task units (MET; multiples of the resting metabolic rate) min/week, or low if the participant performed < 600 MET min/week.

Obesity status was defined according to BMI (not obese: BMI ≥18.5 and < 30 kg/m^2^; obese: BMI ≥30 kg/m^2^) [11]. Hypercholesterolaemia was defined by a total cholesterol level ≥ 5.2 mmol/l (200 mg/dl) [12]. Finally, blood pressure was measured after a 5‐min resting period, with the participant in a seated position. Three measurements, at least 5 min apart, were carried out, and the mean of the second and third measurements was taken. Hypertension was defined as systolic blood pressure ≥ 140 mmHg or diastolic blood pressure ≥ 90 mmHg, or was determined by self‐report of physician diagnosis and current receipt of anti‐hypertensive medication.

Finally, we estimated homeostatic model assessment index for insulin resistance (HOMA‐IR) using the formula developed by Matthews [13] and categorized these into tertiles (low, middle and high).

### Laboratory procedures

Laboratory assessments were performed on venous samples taken in the morning after a minimum of 8 h of fasting. Fasting glucose was measured in plasma using a Cobas^®^ Modular Platform automated analyser and reagents were supplied by Roche Diagnostics (Grenzach‐Whylen, Germany). Serum total cholesterol was measured and HbA_1c_ was measured in whole blood using high‐performance liquid chromatography (D10; BioRad, Munich, Germany), traceable to the Diabetes Control and Complications Trial reference study, as certified by the National Glycohaemoglobin Standardization Programme [14]. All samples were analysed at a single facility, and, for quality assurance, assay results were checked with regular external standards and internal duplicate assays and monitored by BioRad (www.biorad.com) [8].

### Statistical analysis

All statistical analyses were performed using stata 14.0 (StataCorp, College Station, TX, USA). Prevalence and 95% CIs of different hyperglycaemia categories were determined overall. Mean and sd values for continuous variables and proportions for categorical ones were used to describe variables. The association of different hyperglycaemia categories and characteristics of the study population at baseline were estimated using chi‐squared tests.

For the incidence analysis, all‐cause mortality rates were calculated using incidence rates (the number of failures divided by the person‐time per 1000). Time to event was estimated using the day of death and the log‐rank test was used to compare survival function using different factors (sex, age, education level, socio‐economic status, population group, daily smoking habit). To study the association of intermediate hyperglycaemia, using each of the three definitions, with mortality, crude and adjusted hazard ratios (HRs) and 95% CIs were estimated using Cox proportional hazard models. We used the test of proportional hazards assumption (Schoenfeld residuals) in each model. Variables representing potential confounders were selected by theory using the directed acyclic graph (Fig. S1) and three models were used to estimate HRs and their respective 95% CIs. In addition, a sensitivity analysis was conducted by excluding those with a previous type 2 diabetes diagnosis at baseline.

### Ethics

All participants were informed about the objectives of the study and oral informed consent was obtained owing to high illiteracy rates, especially in rural areas. The institutional review board of *Universidad Peruana Cayetano Heredia* in Peru (Code: 51103) and of the London School of Hygiene and Tropical Medicine in the UK approved the protocol for the baseline assessment, and the follow‐up phase was approved by the *Universidad Peruana Cayetano Heredia* institutional review board (Code: 64904).

## Results

A total of 988 individuals were enrolled at baseline. The mean (sd) age was 47.8 (11.9) years, 52.5% were women and the population groups comprised the following: rural: 207 individuals (20.0%); rural‐to‐urban migrant: 583 individuals (59.7%); and urban: 198 individuals (20.3%).

### Characteristics of the study population, stratified by vital status

At baseline, the prevalence rates of intermediate hyperglycaemia were 6% (95% CI 5–8), 12.9% (95% CI 10.9–15.2) and 38.5% (95% CI 35.5–41.6) using the ADA IFG‐based, IEC HbA_1c_‐based, and ADA HbA_1c_‐based definitions, respectively. The prevalence of type 2 diabetes mellitus was 4% (95% CI 3–5) using the ADA FPG‐based definition, whereas using the ADA and IEC HbA_1c_‐based definitions it was 6% (95% CI 5–8). Cross‐table analysis of the ADA IFG‐based and the ADA HbA_1c_‐based definitions of normal glycaemia, intermediate hyperglycaemia and type 2 diabetes, and the respective ADA IFG‐based and IEC HbA_1c_‐based definitions are shown in Table S1.

Some factors such as age, population group, obesity, hypercholesterolaemia and hypertension were associated with intermediate hyperglycaemia using the three definitions. There was an association between gender and intermediate hyperglycaemia using both the ADA IFG‐based and the ADA HbA_1c_‐based definitions, and an association with education level was additionally observed using both the ADA and IEC HbA_1c_‐based definitions (Table 2).

**Table 2:** Baseline characteristics of the study population, stratified according to normal glycaemia, intermediate hyperglycaemia and type 2 diabetes

	ADA IFG‐based definition			ADA HbA1c‐based definition			IEC HbA1c‐based definition		
	Normal glycaemia	Intermediate hyperglycaemia	Type 2 diabetes	Normal glycaemia	Intermediate hyperglycaemia	Type 2 diabetes	Normal glycaemia	Intermediate hyperglycaemia	Type 2 diabetes
	(*n* = 879)	(*n* = 58)	(*n* = 39)	(*n* = 543)	(*n* = 376)	(*n* = 57)	(*n* = 793)	(*n* = 126)	(*n* = 57)
Men, *n* (%)	426 (48.5)	18 (31.0)	20 (51.3)	283 (52.1)	155 (41.2)	26 (45.6)	380 (47.9)	58 (46.0)	26 (45.6)
Age, *n* (%)									
30–39 years	264 (30.0)	11 (19.0)	6 (15.4)	209 (38.5)	65 (17.3)	7 (12.3)	261 (32.9)	13 (10.3)	7 (12.3)
40–49 years	257 (29.2)	15 (25.9)	6 (15.4)	165 (30.4)	102 (25.1)	11 (19.3)	239 (30.1)	28 (22.2)	11 (19.3)
50–59 years	225 (25.6)	24 (41.4)	20 (51.3)	115 (21.2)	128 (34.0)	26 (45.6)	199 (25.1)	44 (34.9)	26 (45.6)
≥60 years	133 (15.1)	8 (13.8)	7 (18.0)	54 (9.9)	81 (21.5)	13 (22.8)	94 (11.1)	41 (32.5)	13 (22.8)
Education level: ≥7 years, *n* (%)	453 (51.6)	30 (52.6)	20 (51.3)	309 (57.0)	170 (45.3)	24 (42.1)	429 (54.2)	50 (39.7)	24 (42.1)
Socio‐economic status, *n* (%)									
Low	386 (43.9)	23 (39.6)	15 (38.4)	215 (39.6)	185 (49.2)	24 (42.1)	340 (42.9)	60 (47.6)	24 (42.1)
Middle	207 (23.6)	19 (32.8)	12 (30.8)	141 (26.0)	83 (22.1)	14 (24.6)	201 (25.4)	23 (18.3)	14 (24.6)
High	286 (32.5)	16 (27.6)	12 (30.8)	187 (34.4)	108 (28.7)	19 (33.3)	252 (31.8)	43 (34.1)	19 (33.3)
Population group, *n* (%)									
Rural	188 (21.4)	4 (6.9)	3 (7.7)	95 (17.5)	85 (22.6)	15 (26.3)	147 (18.5)	33 (26.2)	15 (26.3)
Rural‐urban migrant	533 (60.6)	30 (51.7)	20 (51.3)	362 (66.7)	195 (51.9)	26 (45.6)	499 (62.9)	58 (46.0)	26 (45.6)
Urban	158 (18.0)	24 (41.4)	16 (41.0)	86 (15.8)	96 (25.5)	16 (28.1)	147 (18.5)	35 (27.8)	16 (28.1)
Daily smoking habit, *n* (%)	30 (3.4)	1 (1.7)	2 (5.1)	12 (2.2)	19 (5.1)	2 (3.5)	24 (3.0)	7 (5.6)	2 (3.5)
High alcohol consumption, *n* (%)	82 (9.3)	2 (3.5)	2 (5.1)	49 (9.0)	34 (9.0)	3 (5.3)	72 (9.1)	11 (8.7)	3 (5.3)
Low levels of physical activity, *n* (%)	220 (25.2)	20 (34.5)	12 (31.6)	142 (26.4)	94 (25.2)	16 (28.6)	206 (26.2)	30 (23.8)	16 (28.6)
Obesity, *n* (%)	148 (16.8)	31 (53.5)	17 (43.6)	80 (14.7)	96 (25.5)	20 (35.1)	140 (17.7)	36 (28.6)	20 (35.1)
Hypercholesterolaemia, *n* (%)	265 (30.2)	19 (32.8)	20 (51.3)	149 (27.5)	129 (34.3)	26 (45.6)	230 (29.0)	48 (38.1)	26 (45.6)
Hypertension, *n* (%)	126 (14.4)	16 (27.6)	13 (33.3)	58 (10.7)	82 (21.8)	15 (26.3)	105 (13.3)	35 (27.8)	15 (26.3)
HOMA‐IR index, *n* (%)									
Low	319 (36.7)	3 (5.2)	1 (2.6)	201 (37.2)	113 (30.5)	9 (16.1)	276 (35.1)	38 (30.7)	9 (16.1)
Middle	310 (35.6)	8 (13.8)	4 (10.3)	210 (38.9)	102 (27.5)	10 (17.9)	279 (35.4)	33 (26.6)	10 (17.9)
High	241 (27.7)	47 (81.0)	34 (87.2)	129 (23.9)	156 (42.1)	37 (66.1)	232 (29.5)	53 (42.7)	37 (66.0)

ADA, American Diabetes Association; HOMA‐IR, homeostatic model assessment index for insulin resistance; IEC, International Expert Committee; IFG, impaired fasting glucose.

### Characteristics of the study population, stratified by death during follow‐up

Data on the vital status of 976 participants were retrieved from the *RENIEC* system. A total of 63 deaths were recorded, with an all cause‐mortality rate of 7 per 1000 person‐years (95% CI 5–8). Crude analysis of mortality and the characteristics of the study population showed strong evidence of associations between mortality and male gender, being older, having a lower education level, having a lower socio‐economic status, and having hypertension (Table S2).

### Association between glucose and HbA_1c_ status and overall mortality

Intermediate hyperglycaemia was associated with 10‐year all‐cause mortality using the HbA_1c_‐based definitions in the crude models, with an almost threefold higher rate in comparison to normal HbA_1c_ levels (Kaplan–Meier curves are shown in Fig. S2). However, in the adjusted models, only the ADA HbA_1c_‐based definition was associated with all‐cause mortality; participants with intermediate hyperglycaemia according to this definition had a mortality rate almost twice as high in comparison to those with normal HbA_1c_ levels.

There was no evidence of an association between the ADA IFG‐based definition of intermediate hyperglycaemia and mortality, either in the crude or the adjusted model (Table 3).

**Table 3:** Association between overall mortality and normal, intermediate hyperglycaemia and type 2 diabetes: crude and adjusted models

	Deaths, *n*/*N* (%)	Crude model	Adjusted model 1*	Adjusted model 2^†^	Adjusted model 3^‡^
		HR (95% CI)	HR (95% CI)	HR (95% CI)	HR (95% CI)
ADA IFG‐based definition					
Normal	54/879 (6.1)	1 (Reference)	1 (Reference)	1 (Reference)	1 (Reference)
Intermediate hyperglycaemia	3/58 (5.2)	0.84 (0.26–2.68)	0.85 (0.27–2.74)	1.01 (0.31–3.26)	1.09 (0.33–3.63)
Type 2 diabetes	6/39 (15.4)	**2.62 (1.13–6.08)**	2.14 (0.91–5.00)	2.15 (0.89–5.17)	**2.92 (1.18–7.22)**
ADA HbA1c‐based definition					
Normal	18/543 (3.3)	1 (Reference)	1 (Reference)	1 (Reference)	1 (Reference)
Intermediate hyperglycaemia	34/376 (9.0)	**2.82 (1.59–4.99)**	**1.91 (1.07–3.43)**	**1.81 (1.01–3.27)**	**1.91 (1.03–3.53)**
Type 2 diabetes	11/57 (19.3)	**6.25 (2.95–13.24)**	**4.08 (1.90–8.75)**	**3.82 (1.74–8.37)**	**5.31 (2.34–12.05)**
IEC HbA1c‐based definition					
Normal	36/793 (4.5)	1 (Reference)	1 (Reference)	1 (Reference)	1 (Reference)
Intermediate hyperglycaemia	16/126 (12.7)	**2.92 (1.62–5.28)**	1.46 (0.80–2.66)	1.34 (0.73–2.48)	1.42 (0.75–2.68)
Type 2 diabetes	11/57 (19.3)	**4.53 (2.31–8.91)**	**3.07 (1.55–6.07)**	**2.86 (1.41–5.77)**	**3.89 (1.88–8.05)**

ADA, American Diabetes Association; HR, hazard ratio; IEC, International Expert Committee; IFG, impaired fasting glucose.

The test of proportional hazards assumption was applied and we cannot reject the null hypothesis that the hazards are proportional.

Bold values represent estimates with *P*‐values <0.05 .

* Adjusted for sex, age.

^†^ Adjusted for sex, age, education level, socio‐economic status, population group.

^‡^ Adjusted for sex, age, education level, socio‐economic status, population group, daily smoking, alcohol use, physical activity, hypercholesterolaemia, hypertension and obesity.

However, although the association between type 2 diabetes and 10‐year mortality was significant in the adjusted models using all three definitions, the strength of association varied from an HR of 2.92 (95% CI 1.18–7.22) using the FPG‐based ADA definition, to an of HR 5.31 (95% CI 2.34–12.05) using the HbA_1c_‐based ADA definition.

In the sensitivity analysis, which excluded those with a previous diagnosis of type 2 diabetes, the results were consistent, with slight changes in HR estimates (Table S3).

### Association between glucose and HbA_1c_ status and overall mortality, stratified by population group

The analysis according to population group showed an association between intermediate hyperglycaemia and all‐cause mortality using the ADA HbA_1c_‐based definition in the rural and rural‐urban migrant groups, but not in the urban group. The association in the rural group was four times greater, while the association in the rural‐to‐urban migrant group was 2.5 times greater. Using the IEC HbA_1c_‐based definition, the association was significant only in the rural‐to‐urban migrant group (Table 4).

**Table 4:** Association between overall mortality and normal glycaemia, intermediate hyperglycaemia and type 2 diabetes according to rural, rural‐to‐migrant and urban populations: crude analysis

	Rural (*N*=195) HR (95% CI)	Rural‐urban migrant (*N* =583) HR (95% CI)	Urban (*N* =198) HR (95% CI)
ADA IFG‐based definition			
Normal	1 (Reference)	1 (Reference)	1 (Reference)
Intermediate hyperglycaemia	‐	1.27 (0.30–5.33)	0.72 (0.09–5.69)
Type 2 diabetes	5.16 (0.69–38.85)	1.91 (0.46–8.03)	3.41 (0.92–12.60)
ADA HbA1c‐based definition			
Normal	1 (Reference)	1 (Reference)	1 (Reference)
Intermediate hyperglycaemia	**4.35 (1.21–15.60)**	**2.52 (1.20–5.34)**	2.17 (0.56–8.40)
Type 2 diabetes	**9.68 (1.21–15.61)**	**4.87 (1.57–15.11)**	**5.69 (1.15–28.21)**
IEC HbA1c‐based definition			
Normal	1 (Reference)	1 (Reference)	1 (Reference)
Intermediate hyperglycaemia	1.22 (0.34–4.37)	**4.34 (1.97–9.60)**	2.94 (0.82–10.40)
Type 2 diabetes	**3.97 (1.26–12.48)**	**4.23 (1.44–12.46)**	**4.81 (1.20–19.25)**

ADA, American Diabetes Association; HR, hazard ratio; IEC, International Expert Committee; IFG, impaired fasting glucose.

Bold values represent estimates with *P*‐values <0.05 .

The association between type 2 diabetes and mortality was significant using both the ADA and the IEC HbA_1c_‐based definitions in the three groups; however, the association in the rural group was 9.6 times greater, whereas in the rural‐to‐urban migrant and the urban groups it was 4.8 and 5.7 times greater, respectively, using the ADA HbA_1c_‐based definition.

## Discussion

Given that there are several different definitions of intermediate hyperglycaemia, and that these use arbitrary cut‐off points, further studies of these definitions and their associations with patient‐important outcomes are relevant. The present study adds to the literature by assessing mortality rates among individuals with intermediate hyperglycaemia using population‐based data. The prevalence of intermediate hyperglycaemia according to the three studied definitions varied from 5.9% to 38.5%. The study also found that individuals with HbA_1c_ levels between 39 and 46 mmol/mol (5.7 and 6.4%) had a higher rate of mortality in comparison to people with HbA_1c_ levels < 39 mmol/mol (5.7%) in this cohort from Peru, at 10‐year follow‐up. The IEC HbA_1c_‐based definition was associated with an increased risk of mortality, although this was not statistically significant. There was no evidence of an association between the ADA IFG‐based definition of intermediate hyperglycaemia and mortality, but such an observation was observed when using the ADA HbA_1c_‐based definition, although participants with intermediate hyperglycaemia according to the latter definition did have a somewhat more favourable cardiometabolic risk profile, especially with regard to obesity.

Our findings differ from those of a systematic review and meta‐analysis by Huang *et al.* [5] who reported that intermediate hyperglycaemia defined by fasting glucose level was associated with all cause‐mortality, but they did not find an association using the HbA_1c_ definitions [5]. Similar to our results, however, a previous systematic review by the same author group [15], as well as a study from Taiwan [6], reported no association between intermediate hyperglycaemia, assessed using the ADA IFG‐based definition, and all‐cause mortality and cardiovascular disease and/or diabetes mortality. It is important to mention that, in the recent systematic review [5], only the studies conducted in the Netherlands and Finland, and not in other countries, reported a strong association between ADA IFG‐defined intermediate hyperglycaemia and all‐cause mortality. In addition, only studies from the USA, Asia, Europe and Australia were included in the systematic review, and it did not include, for example, individuals with different sociodemographic and lifestyle characteristics from those living in Latin America. Genetics might play a role in the difference among findings worldwide: the admixture among Peruvians is very high, with many groups sharing common Native American ancestry [16], whereas the European ancestry component is relatively small (<10%) [17].

The correlation between glucose and HbA_1c_ levels might also play a role in the differences between the findings of the present study when compared to those of the previous systematic reviews as these variables are affected by factors such as high altitude [18], environmental temperature [19], or the characteristics of the tests themselves [20]. In addition, high altitude could explain the fact that the HRs for the associations between both intermediate hyperglycaemia and type 2 diabetes with mortality were higher in the rural population in comparison to the rural‐to‐urban migrant and urban populations; a previous study in Peru found that 53 mmol/mol (6.5%) of HbA_1c_ was correlated to glucose levels of 6.6. mmol/l at sea levels and 14.8 mmol/l at high altitude. Furthermore, in India, a lower HbA_1c_ threshold has been used to define diabetes [45 mmol/mol (6.3%)], based on the glucose torelance test [21], which highlights the fact that some participants in the present study might have been misclassified as having intermediate hyperglycaemia using HbA_1c_, when in reality, they have type 2 diabetes mellitus.

As mentioned in the introduction, the association between type 2 diabetes mellitus and all‐cause mortality is well known; however, the estimations of the associations in the present study (HRs of 2.92–5.30 according to the different definitions) are higher in comparison to those in previous studies. The relatively recent systematic review of all‐cause mortality among individuals with type 2 diabetes in Latin America found a relative risk of between 2.26 (95% CI 1.36–3.74) and 2.49 (95% CI 1.96–3.15) using a composite definition and self‐reported diagnosis, respectively [7]. The highest association observed in the systematic review was an HR of 6.64 (95% CI 1.94–22.8) in a cohort in Brazil with 13 years of follow‐up [22], and another high value was the HR of 4.38 (95% CI 3.43–5.59) reported in a cohort in Mexico with a 15‐year follow‐up [23]. These are greater than the associations of mortality and type 2 diabetes found in Sweden (adjusted HR 1.15, 95% CI 1.14–1.16) in a cohort with a 4‐year follow‐up, [4] and in England (adjusted HR 2.19, 95% CI 2.16–2.21) in a cohort followed for 17 years [24]. The differences between high‐income countries and countries in Latin America may be the result of disparities in access to healthcare, insurance coverage, and the quality of healthcare received between such countries [25].

The findings of the present study are relevant as individuals with HbA_1c_ between 39 and 46 mmol/mol (5.7% and 6.4%) had higher mortality than those with normal HbA_1c_ levels, showing that the former probably require special management to prevent negative outcomes. However, a systematic review including individuals with intermediate hyperglycaemia found that screening and treatment strategies to prevent type 2 diabetes mellitus had limitations because even if both short‐ (6 months) and long‐term (up to 7 years) interventions delayed the onset of type 2 diabetes, many individuals might have been incorrectly classified as having intermediate hyperglycaemia [26]. Nowadays, lifestyle interventions are the standard of care for impaired glucose tolerance [27], but there is limited evidence for these in the management of IFG or intermediate hyperglycaemia defined according to HbA_1c_ levels. Moreover, the management of intermediate hyperglycaemia with drugs is still controversial in terms of efficacy and cost [28, 29].

Finally, previous studies have found that the prevalence of intermediate hyperglycaemia increases four times using the ADA definitions (based on IFG and/or HbA_1c_) [6, 30], which could imply a huge challenge to health systems. Similarly, another study in Peruvian population found that the magnitude of regression to normal glucose/HbA_1c_ levels was much higher than progression towards type 2 diabetes after 2.5 years of follow‐up [31]. So, even if we know that the ADA HbA_1c_‐based definition of intermediate hyperglycaemia is associated with a high mortality rate, the available evidence is not clear on how to manage this risk to prevent progression to type 2 diabetes and/or mortality.

To date, nearly all efforts to synthesize the evidence regarding intermediate hyperglycaemia have concentrated on high‐income countries. In the present study, we show that intermediate hyperglycaemia using the ADA HbA_1c_‐based definition may be important for estimating the rate of mortality in resource‐constrained settings, contributing to the knowledge in this area in a poorly studied population.

The study has some limitations that should be noted. First, all‐cause mortality was used as the outcome because information about cause of death was not available using the *RENIEC* records. Second, a potential time gap between the date of death and its registration in *RENIEC* is possible and, which could have led us to underestimate the incidence of death in our population. Additionally, the factors identified as associated with mortality (Table S3) were not adjusted for confounders. Third, we could not include the WHO definition of IFG [FPG 6.1–6.9 mmol/l (110–125 mg/dl)] because we did not have cases with that definition in our sample. Fourth, an oral glucose tolerance test was not performed at baseline for logistical and budget reasons. However, we based our definition on the use of fasting glucose and HbA_1c_ as in previous studies. Fifth, our study population was not nationally representative; however, it included different population groups (rural, rural‐to‐urban migrants and urban) from specific areas of Peru. Finally, HbA_1c_ is usually a specific measurement by which to diagnose and monitor diabetes, but other conditions could increase HbA_1c_ levels, such as iron deficiency anaemia, a condition that might be more common in rural areas or in people of low socio‐economic status. Additionally, other less common conditions in Peru could affect HbA_1c_ levels, such as kidney failure, liver disease, sickle cell disease and thalassaemia [9].

In conclusion, intermediate hyperglycaemia using the ADA HbA_1c_‐based definition was associated with an increased mortality rate in cohort from Peru with a 10‐year follow‐up. HbA_1c_ would appear to be a variable associated with mortality in this Peruvian population. Further research is needed, however, on how to prevent progression to type 2 diabetes and/or mortality in this population.

## Ethical Approval

The study was approved by the Medical Ethics Committee of [INSTITUTION], and informed consent was obtained from all participants. This research study was conducted in accordance with the guidelines of the Declaration of Helsinki.

## Funding sources

The study was funded by a Strategic Award from the Wellcome Trust‐Imperial College Centre for Global Health Research (100693/Z/12/Z) and the Imperial College London Wellcome Trust Institutional Strategic Support Fund (Global Health Clinical Research Training Fellowship) (294834/Z ISSF ICL). R.M.C.‐L. is supported by a Wellcome Trust International Training Fellowship (214185/Z/18/Z). M.L.‐P. receives funding from the Swiss Excellence Government Scholarship (2018.0698). A.B.‐O. (103994/Z/14/Z) and J.J.M. (074833/Z/04/Z, 205177/Z/16/Z) are supported by the Wellcome Trust. J.J.M. acknowledges receiving additional support from the Alliance for Health Policy and Systems Research (HQHSR1206660), the Fogarty International Centre (R21TW009982, D71TW010877), Grand Challenges Canada (0335‐04), the International Development Research Centre Canada (106887, 108167), the Inter‐American Institute for Global Change Research (IAI CRN3036), the Medical Research Council (MR/P008984/1, MR/P024408/1, MR/P02386X/1), the National Cancer Institute (1P20CA217231), the National Heart, Lung and Blood Institute (HHSN268200900033C, 5U01HL114180, 1UM1HL134590), the National Institute of Mental Health (1U19MH098780), the Swiss National Science Foundation (40P740‐160366), and the World Diabetes Foundation (WDF15‐1224).

## Competing interests

None declared.

## Supporting information


**Figure S1.** Directed acyclic graph.
**Figure S2.** Kaplan–Meier curves by ADA glucose status, IEC HbA_1c_ status and ADA HbA_1c_ status.
**Table S1.** Cross tables of the different definitions: (a) glucose ADA vs HbA_1c_ ADA, (b) glucose ADA vs HbA_1c_ IEC.
**Table S2.** Baseline characteristics of the study population according to death during follow‐up.
**Table S3. **Sensitivity analysis.
