# Supplementary material for: Intermediate hyperglycaemia and 10‐year mortality in resource‐constrained settings: the PERU MIGRANT Study
Source: Diabet Med. 2020 Apr 3;37(9):1519–27. doi: 10.1111/dme.14298 (PMC7649719; doi:10.1111/dme.14298)
Supplement: Supplementary file 1 — Figure S1. Directed acyclic graph. Figure S2. Kaplan–Meier curves by ADA glucose status, IEC HbA1c status and ADA HbA1c status. Table S1. Cross tables of the different definitions: (a) glucose ADA vs HbA1c ADA, (b) glucose ADA vs HbA1c IEC. Table S2. Baseline characteristics of the study population according to death during follow‐up. Table S3. Sensitivity analysis. [file DME-37-1519-s001.docx]

## Supplementary Material 1: Directed Acyclic Graph


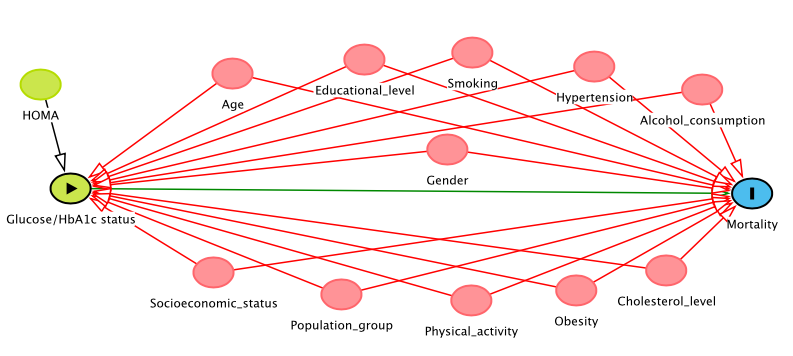


## Supplementary Material 2: Cross tables of the different definitions

1. **Glucose ADA vs HbA1c ADA**

|  | | **Glucose ADA** | | |
| --- | --- | --- | --- | --- |
|  |  | Normal | Intermediate hyperglycaemia | Type 2 diabetes mellitus |
| **HbA1c ADA** | Normal | 524 (96.5%) | 19 (3.5%) | 0 (0%) |
|  | Intermediate hyperglycaemia | 341 (90.7%) | 35 (9.3%) | 0 (0%) |
|  | Type 2 diabetes mellitus | 14 (24.6%) | 4 (7.0%) | 39 (68.4%) |

HbA1c: Glycated Haemoglobin, ADA: American Diabetes Association, IEC: International Expert Committee,

1. **Glucose ADA vs HbA1c IEC**

|  | | **Glucose ADA** | | |
| --- | --- | --- | --- | --- |
|  |  | Normal | Intermediate hyperglycaemia | Type 2 diabetes mellitus |
| **HbA1c IEC** | Normal | 759 (95.7%) | 34 (4.3%) | 0 (0%) |
|  | Intermediate hyperglycaemia | 106 (84.1%) | 20 (15.9%) | 0 (0%) |
|  | Type 2 diabetes mellitus | 14 (24.6%) | 4 (7.0%) | 39 (68.4%) |

HbA1c: Glycated Haemoglobin, ADA: American Diabetes Association, IEC: International Expert Committee,

## Supplementary Material 3: Baseline characteristics of the study population according to death during follow-up

|  | **Alive** | **Dead** | **p-value** |
| --- | --- | --- | --- |
|  | **(n = 913)** | **(n = 63)** |  |
| **Sex** |  |  |  |
| Female | 490 (95.7%) | 22 (4.3%) | **0.004** |
| Male | 423 (91.2%) | 41 (8.8%) |  |
| ***Age*** |  |  |  |
| 30 – 39 years | 279 (99.3%) | 2 (0.7%) | **< 0.001** |
| 40 – 49 years | 272 (97.8%) | 6 (2.2%) |  |
| 50 – 59 years | 253 (94.1%) | 16 (5.9%) |  |
| 60+ years | 109 (73.7%) | 39 (26.4%) |  |
| ***Education level*** |  |  |  |
| < 7 years | 428 (90.9%) | 43 (9.1%) | **0.001** |
| 7+ years | 483 (96.0%) | 20 (4.0%) |  |
| ***Socioeconomic status*** |  |  |  |
| Low | 386 (91.0%) | 38 (9.0%) | **0.01** |
| Middle | 230 (96.6%) | 8 (3.4%) |  |
| High | 297 (94.6%) | 17 (5.4%) |  |
| ***Population group*** |  |  |  |
| Rural | 177 (90.8%) | 18 (9.2%) | 0.18 |
| Migrant | 551 (94.5%) | 32 (5.5%) |  |
| Urban | 185 (93.4%) | 13 (6.6%) |  |
| ***Daily smoking*** |  |  |  |
| No | 880 (93.5%) | 61 (6.5%) | 0.95 |
| Yes | 31 (93.9%) | 2 (6.1%) |  |
| ***Alcohol use*** |  |  |  |
| Low | 834 (93.7%) | 56 (6.3%) | 0.52 |
| High | 79 (91.9%) | 7 (8.1%) |  |
| ***Physical activity*** |  |  |  |
| Moderate/high levels | 667 (93.2%) | 49 (6.8%) | 0.35 |
| Low levels | 239 (94.8%) | 13 (5.2%) |  |
| ***Obesity*** |  |  |  |
| No | 727 (93.2%) | 53 (6.8%) | 0.39 |
| Yes | 186 (94.9%) | 10 (5.1%) |  |
| ***Hypercholesterolemia*** |  |  |  |
| No | 623 (92.9%) | 48 (7.2%) | 0.13 |
| Yes | 290 (95.4%) | 14 (4.6%) |  |
| ***Hypertension*** |  |  |  |
| No | 786 (95.9%) | 34 (4.1%) | **< 0.001** |
| Yes | 126 (81.3%) | 29 (18.7%) |  |
| ***HOMA Index*** |  |  | 0.02 |
| First tertile | 293 (90.7%) | 30 (9.3%) |  |
| Second tertile | 309 (95.9%) | 13 (4.1%) |  |
| Third tertile | 304 (94.4%) | 18 (5.6%) |  |

* P-value was estimated using the Log-rank test

## Supplementary material 4. Kaplan-Meier Curves

**American Diabetes Association - glucose**

**International Expert Committee - HbA1c**

**American Diabetes Association - HbA1c**

## Supplementary Material 5. Sensitivity Analysis

|  | **Glucose - ADA** | **HbA1c - ADA** | **HbA1c - IEC** |
| --- | --- | --- | --- |
|  |  |  |  |
| **Crude** |  |  |  |
| Normal | 1 (Reference) | 1 (Reference) | 1 (Reference) |
| T2DM (no previously known diabetes) | **2.65 (1.06 – 6.63)** | **6.48 (2.99 – 14.05)** | **4.69 (2.33 – 9.47)** |
| **Adjusted model 3*** |  |  |  |
| Normal | 1 (Reference) | 1 (Reference) | 1 (Reference) |
| T2DM (no previously known diabetes) | 2.62 (0.99 – 6.94) | **5.01 (2.16 – 11.60)** | **3.69 (1.74 – 7.82)** |
| **Crude** |  |  |  |
| Intermediate hyperglycemia | 1 (Reference) | 1 (Reference) | 1 (Reference) |
| T2DM (no previously known diabetes) | 3.14 (0.75 – 13.14) | **2.29 (1.13 – 4.65)** | 1.59 (0.72 – 3.52) |
| **Adjusted model 3*** |  |  |  |
| Intermediate hyperglycemia | 1 (Reference) | 1 (Reference) | 1 (Reference) |
| T2DM (no previously known diabetes) | 9.84 (0.67 – 144.07) | **2.65 (1.24 – 5.67)** | **2.88 (1.13 – 7.33)** |

HbA1c: Glycated Haemoglobin, ADA: American Diabetes Association, IEC: International Expert Committee, T2DM Type 2 Diabetes Mellitus.
