# Supplementary material for: Intermediate hyperglycaemia and 10‐year mortality in resource‐constrained settings: the PERU MIGRANT Study
Source: Diabet Med. 2020 Apr 3;37(9):1519–27. doi: 10.1111/dme.14298 (PMC7649719; doi:10.1111/dme.14298)
Supplement: Supplementary file 2 [file DME-37-1519-s002.docx]

Supplementary references

1. IPAQ Research Committee . Karolinska Institutet; Stockholm: 2005. Guidelines for Data Processing and Analysis of the International Physical Activity Questionnaire L (IPAQ)—short and Long Forms.<https://sites.google.com/site/theipaq/scoring-protocol> [cited 2015 April 24].

2. James PA, Oparil S, Carter BL, Cushman WC, Dennison-Himmelfarb C, Handler J, et al. 2014 evidence-based guideline for the management of high blood pressure in adults: report from the panel members appointed to the Eighth Joint National Committee (JNC 8). JAMA. 2014;311(5):507-20.

3. Matthews DR, Hosker JP, Rudenski AS, Naylor BA, Treacher DF, Turner RC. Homeostasis model assessment: insulin resistance and beta-cell function from fasting plasma glucose and insulin concentrations in man. Diabetologia. 1985;28(7):412-9.

4. Registro Nacional de Identificación y Estado Civil: Peru; 2019 [Available from: <https://www.reniec.gob.pe/portal/intro.htm>.

5. Miranda JJ, Gilman RH, Smeeth L. Differences in cardiovascular risk factors in rural, urban and rural-to-urban migrants in Peru. Heart. 2011;97(10):787-96.

6. Xue L, Liang H, Jiang X. Circannual temperature-related variation in hemoglobin A1c is unlikely to affect its use as a diagnostic test for type 2 diabetes. Clin Lab. 2012;58(5-6):481-8.

7. Yates T, Haffner SM, Schulte PJ, Thomas L, Huffman KM, Bales CW, et al. Association between change in daily ambulatory activity and cardiovascular events in people with impaired glucose tolerance (NAVIGATOR trial): a cohort analysis. Lancet. 2014;383(9922):1059-66.

8. Li G, Zhang P, Wang J, An Y, Gong Q, Gregg EW, et al. Cardiovascular mortality, all-cause mortality, and diabetes incidence after lifestyle intervention for people with impaired glucose tolerance in the Da Qing Diabetes Prevention Study: a 23-year follow-up study. Lancet Diabetes Endocrinol. 2014;2(6):474-80.

9. Kanat M, DeFronzo RA, Abdul-Ghani MA. Treatment of prediabetes. World J Diabetes. 2015;6(12):1207-22.

10. Cardenas MK, Miranda JJ, Beran D. Delivery of Type 2 diabetes care in low- and middle-income countries: lessons from Lima, Peru. Diabet Med. 2016;33(6):752-60.

11. Tarqui-Mamani C, Sanchez-Abanto J, Alvarez-Dongo D, Espinoza-Oriundo P, Jordan-Lechuga T. Prevalencia de anemia y factores asociados en adultos mayores peruanos. Revista Peruana de Medicina Experimental y Salud Publica. 2015;32(4):687-92.

12. WHO. Definition and diagnosis of diabetes mellitus and intermediate hyperglycaemia. Geneva: World Health Organization; 2006.
